# Symptomatic individuals with Lumbar Disc Degeneration use different anticipatory and compensatory kinematic strategies to asymptomatic controls in response to postural perturbation

**DOI:** 10.1016/j.gaitpost.2021.03.037

**Published:** 2022-05

**Authors:** Janet A. Deane, Adrian K.P. Lim, Andrew T.M. Phillips, Alison H. McGregor

**Affiliations:** a MSK LAB, Imperial College London, London, United Kingdom; b Structural Biomechanics, Department of Civil and Environmental Engineering, Imperial College London, United Kingdom; c Imaging Department, Charing Cross Hospital, Imperial College Healthcare NHS Trust, London, United Kingdom; d School of Health Sciences, The University of Manchester and University of Manchester NHS Foundation Trust, Manchester, United Kingdom

**Keywords:** Back pain, Lumbar degeneration, Kinematics, Postural control, Motion capture

## Abstract

**Background:**

Lumbar Disc Degeneration (LDD) is associated with recurrent low back pain (LBP) (symptomatic). However, in some instances of LDD, people do not experience LBP (asymptomatic).

**Research question:**

As a step towards understanding why some people with LDD experience LBP and others do not, the primary aim of this study was to examine differences in anticipatory (APA) and compensatory postural adjustments (CPA), between symptomatic LDD patients (LDD pain) and asymptomatic LDD controls (LDD no pain) during postural perturbation. The secondary aim was to determine simultaneous differences in mental health, disability and quality of life status.

**Methods:**

3 T MRI was used to acquire T2 weighted images (L1-S1) from LDD no pain (n = 34) and LDD pain groups (n = 34). In this observational study, responses to predicted and unpredicted forward perturbations were examined using three dimensional motion capture. A Mann Whitney *U* test was conducted to examine group differences in sagittal spine and lower limb kinematics (integrated angular displacements during four established APA and CPA time intervals), anxiety, depression, disability and quality of life.

**Results:**

The LDD pain group exhibited lower hip and knee displacements (p = 0.049−0.040) than the LDD no pain group during predicted and unpredicted perturbation. The LDD pain group also exhibited higher compensatory lumbar displacement than the LDD no pain group (p = 0.040−0.005) in the predicted condition but there was no difference observed in the unpredicted condition. The LDD pain group experienced higher levels of depression, anxiety and disability (p < 0.0001) and lower quality of life (p = 0.0001) than LDD controls.

**Significance:**

Symptomatic LDD patients are different from LDD controls; they exhibit different kinematic strategies, levels of disability, anxiety, depression and quality of life. Effective care may benefit from evaluating and targeting these differences.

## Highlights

• Lumbar Disc Degeneration (LDD) is commonly associated with low back pain.
• Symptomatic LDD patients exhibit different postural strategies to LDD controls.
• This strategic difference is observed within the trunk and lower limbs.
• Effective care may benefit from evaluating and targeting these differences.

## 1 Introduction

It is acknowledged that the future of effective health care will be determined by targeted management; the right person receiving the right care [1]. Current treatments for chronic low back pain (LBP) are largely ineffective [2,3]. Therefore, in order to target care sensibly, or to establish risk or future prognosis, there is a pressing need to understand the specific differences between asymptomatic controls and symptomatic patients with associated spinal pathology.

Functional task analysis has been used to successfully discriminate between asymptomatic controls and LBP patients using three-dimensional motion capture (3DMC). However, findings seem contradictory and inconclusive due to the established heterogeneity of this population [[4], [5], [6], [7], [8], [9]]. In addition, most LBP kinematic studies use small sample sizes (10–23 subjects per group group) [[4], [5], [6]], single segmented models of the spine [6] or do not consider the lower limb [10]. Therefore, this current study builds upon this by evaluating the spine and bilateral lower limb kinematics using a 10 segment model of the spine, pelvis and lower limbs within a larger, relatively homogeneous LBP cohort of symptomatic and asymptomatic people with Lumbar Disc Degeneration (LDD) [[10], [11], [12]].

Impaired postural control is frequently associated with LBP [13]. Therefore, the delivery of external predicted and unpredicted perturbations beneath the feet is often used to examine changes in postural control, including anticipatory (APA) and compensatory postural adjustments (CPAs) between LBP patients and healthy controls [14,15]. APAs occur prior to any predicted perturbation event in order to minimise disequilibrium or falling [16,17], while CPAs restore equilibrium following predicted and unpredicted events [18,19].

The primary objective of this study was to examine differences in sagittal trunk and lower limb displacements between symptomatic LDD patients (LDD pain) and asymptomatic LDD controls (LDD no pain) in the APA and CPA phases of predicted and unpredicted forward postural perturbation using 3DMC. Since patients with LBP commonly experience psychological changes and disability, the secondary objective was to simultaneously examine these differences between the same groups using validated self-report questionnaires.

## 2 Methods

### 2.1 Participants

Ethical approval was granted from the NHS Health Research Authority (NRES Committee London, Stanmore, REC reference number: 13/LO/0793). A priori analysis confirmed that a minimum total sample size of fifty-eight subjects (29 per group) would be required to deliver sufficient power (0.80) (α = 0.05) (G*Power Statistical Power Analyses, Dusseldorf, Germany). Patients were recruited from primary and secondary care and healthy controls through local advertisement as part of a larger study and provided informed consent (n = 97). Since preliminary findings determined that significant differences in motor control lay between LDD pain and LDD no pain groups, these groups (sixty-eight participants (34 per group) became the primary focus of this study. LDD is common, with a prevalence of 40 % of subjects under 30 years of age, increasing to 90 % at 50–55 years [20]. Therefore, participants of ≥ 30 years were included in this study (see Table 1 for strict inclusion and exclusion criteria). Participants were identified as ‘LDD’ if they had modified Pfirrmann grade of ≥6 at one or more lumbar levels [21] and as ‘LBP’ if they experienced recurrent LBP for ≥ 3 months duration. Demographics (sex, age, weight, height and BMI) were obtained. Self-reported clinical outcomes included an 11-point Numerical Rating Scale (NRS) [22], the Short Form 36, Version 2 (SF-36) [23], the Oswestry Disability Index (ODI) [24] and the Hospital Anxiety and Depression Scale (HADS) [25] to assess pain related changes during the trial, quality of life, disability and anxiety and depression, respectively.

**Table 1:** Inclusion and exclusion criteria.

	Inclusion Criteria	Exclusion Criteria
Healthy Controls	≥ 30 years Evidence of LDD without neural compression on MRI No low back pain No recurrent history of LBP No episodes of LBP lasting ≥ 3 months duration	Spinal surgery Malignancy Spondylolisthesis Peripheral neuropathy with loss of sensation Systemic or spinal infection Neurological disease or balance disorder Disorders affecting pain perception Significant cardiovascular or metabolic disease Severe musculoskeletal deformity (scoliosis, osteoporosis, Paget’s disease, fracture) Spinal surgery or major surgery within three months prior to testing MRI contraindicated Perturbation contraindicated
Patients	≥ 30 years MRI as part of routine NHS care Evidence of LDD without neural compression on MRI Recurrent LBP (central/ unilateral) of ≥ 3 months duration	

### 2.2 Experimental procedures

A 3 T Verio MRI scanner (Siemens Medical Systems, Erlangen, Germany) was used to acquire supine T2 weighted sagittal lumbar spine images (L1-L5/S1) (TR = 3000 ms, TE=92 ms, 15 slices, 4 mm slice width with 0.5 mm gap) from healthy controls and patients as part of their consented involvement in the study and routine NHS care respectively.

**Fig. 1:**
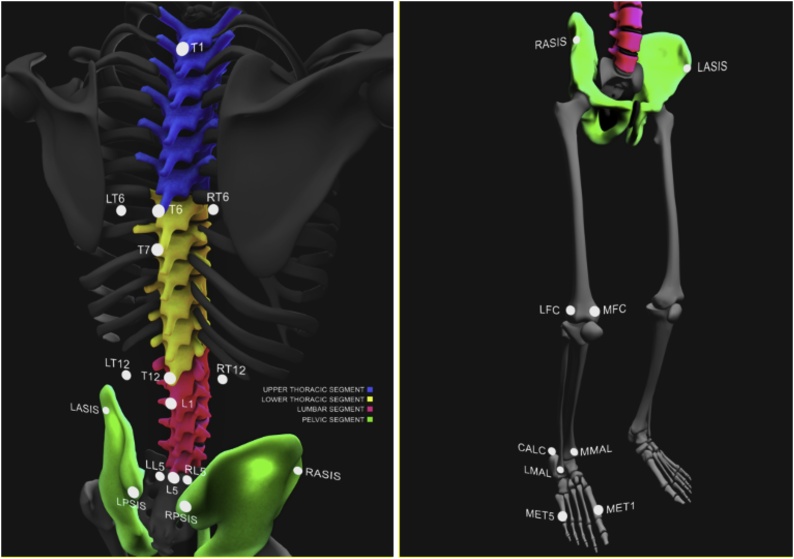
'Imperial Spine' marker positioning. ‘Imperial Spine’ markers positioned on the dorsal spine (below, left) and ventral lower limbs (below, right). The upper thoracic spine segment (left, blue) is represented by markers placed on T1 and T6 spinous processes and LT6 and RT6 markers placed 2.5 cm to the right (RT) and left (LT) of the T6 spinous process. The lower thoracic segment (left, yellow) is represented by markers placed on T7 and T12 spinous processes and the LT12 and RT12 markers placed 2.5 cm to the right and left of the T12 spinous process. The lumbar segment (left, red) is represented by L1 and L5 and the LL5 and RT5 markers placed 2.5 cm to the right and left of L5 spinous process. Pelvic (RASIS (right anterior superior iliac spine), LASIS (left anterior superior iliac spine), LPSIS (left posterior superior iliac spine) and RPSIS (right posterior superior iliac spine)), thigh (right (RFC) and left (LFC) femoral condyles), shank (medial (MMAL) and lateral (LMAL) malleoli), foot and ankle (Head of 5th metatarsal (MET5), Head of 1st metatarsal (MET1) and Calcaneus (CALC)) markers are also highlighted.

The ‘Imperial Spine’ marker set was used to assess sagittal trunk and lower limb kinematics [26,27]. 18 single spherical retro-reflective markers (14 mm diameter) (at T1, T7 and L1 spinous processes and bilateral anterior and posterior iliac spines, lateral and medial femoral condyles, lateral and medial malleoli, head of 1^st^ and 5^th^ metatarsals and calcanei), 3 triads (3 strips of 3 single markers fixed in linear orientation on rubber strips at T6, T12 and L5) (Fig. 1). In addition, one pelvic cluster [28] and two rigid thigh and shank clusters [29] were applied using double-sided tape.

The spine, pelvis and bilateral lower limbs were modelled as 10 rigid segments according to identifiable anatomical landmarks. The upper thoracic (UT) segment (T1-T6) was defined with its origin in T6, vertical axis from T6 to T1 (+y) and horizontal axis through T6 (+z to the right). The lower thoracic (LT) segment (T7-T12) was defined with its origin in T12, vertical axis from T12 to T7 (+y) and horizontal axis through T12 (+z to the right). The lumbar (L) segment (L1-L5) was defined with its origin in L5, vertical axis from L5 to L1 (+y) and horizontal axis through L5 (+z to the right).

Pelvic, thigh, shank and foot local co-ordinate systems were defined [30,31] and reconstructed from joint centres and anatomical landmarks on the pelvis and lower limb. The origins of each segment lay at the joint centre. Harrington regression equations were used to predict the hip joint centres using pelvic anatomical landmarks (ASIS and PSIS) [32]. The knee and ankle joint centres were defined as the midpoint between the medial and lateral epicondyles and the medial and lateral malleolus respectively [30].

The anatomical frames of the pelvis, thigh and shank were then referenced to the corresponding technical frames in the static calibration trial such that anatomical markers (ASIS, PSIS, MFC, LFC, LMAL, MMAL) could be removed prior to dynamic trials, permitting freedom of movement. All trials were recorded at 100 Hz using a 10-camera 3DMC system (Vicon Nexus (T160), Oxford Metrics Ltd., Oxford, UK) [33].

**Fig. 2:**
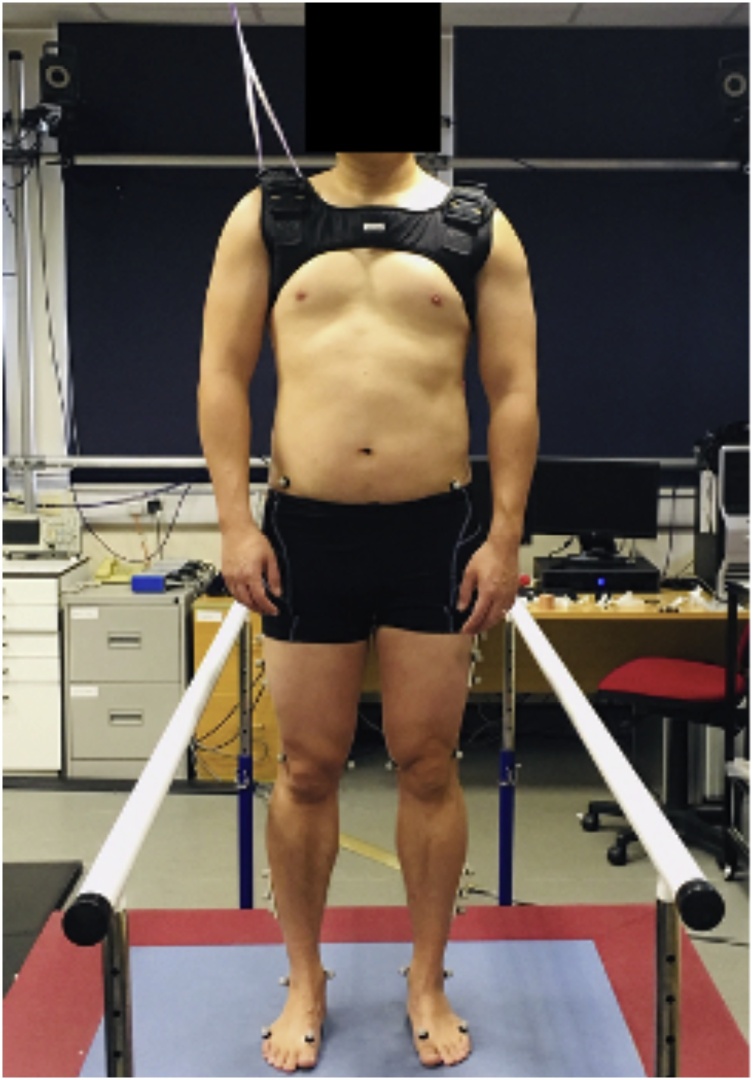
Experimental set up. One representative participant wearing a bespoke harness and standing on the perturbation platform with the ‘Imperial Spine’ marker set applied.

Following application of the marker set, participants stood barefoot with feet hip width apart in the centre of the perturbation platform (Fig. 2) [34]. Participants were instructed that perturbations would comprise of predictable and unpredictable forward perturbations; if predicted, the participant was advised on the precise timing and direction of the perturbation using auditory cues, if unpredicted, the participant was unable to predict the precise timing and perturbation direction. Participants were faced away from the computer as perturbations were triggered. Throughout the experiment participants wore a bespoke safety harness and had access to handrails. Participants received three repeated predictable and unpredictable forward perturbations, the magnitude of which was designed to permit feet-in-place responses (40 mm in 0.2 s, average acceleration 1.97 m/s^2^) with the acceleration profile designed to simulate a train on the London Underground [34]. The conditions were presented in the same order to each subject. Standardardised verbal instruction and a foot template was designed to maximise consistency of the base of support adopted by each participant.

### 2.3 Data processing

The onsets of kinematic signals were synchronised with the onset of platform perturbation. An in-house constructed accelerometer (1000 Hz) was attached directly to the platform. The onset of perturbation was verified by a combination of visual verification and a computer algorithm reflecting the Shewart protocol [35]. In the event of accelerometer failure (as occurred in 4 trials), the onset was determined using the acceleration of a retroreflective platform marker fixed to the platform.

The baseline joint displacement was calculated within the -500 to −450 ms window prior to perturbation (0 ms). This baseline was subtracted from all outputs in order to ensure that the results were due to true differences and not differences in baseline [36]. Since each APA and CPA time interval represented 150 ms and the baseline window represented 50 ms, three times the integral of the baseline activation was subtracted from the integral of joint displacement or total range of movement [36].

**Fig. 3:**
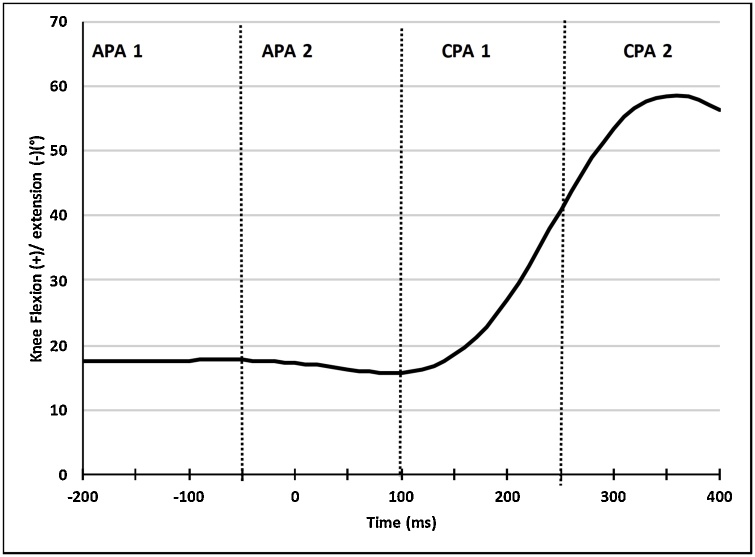
Representative trace of knee displacement. This figure represents the original data from right knee of one healthy subject. The peak amplitude was defined as the maximum amplitude of angular displacement (°) following perturbation at time =0 ms. Time epochs were defined as −200 ms to −50 ms (APA1), −50 ms to +100 ms (APA2), +100 ms to 250 ms (CPA1) and 250 ms–400 ms (CPA2).

Differences in kinematic strategy were defined as differences in integrated sagittal spine (upper thoracic, lower thoracic, lumbar) and/or lower limb (hip, knee and ankle) displacement waveforms between groups during specific APA and CPA phases. Since there is a known 50 ms electromechanical delay between the onset latency of skeletal muscle and the tension development within a muscle [37], each phase was shifted forward by 50 ms [36]. The following phases were examined: −200 ms to −50 ms (APA1), −50 ms to +100 ms (APA2), +100 ms to 250 ms (CPA1) and 250 ms–400 ms (CPA2) [36] (Fig. 3).

There was no learning effect observed with respect to the three repeated perturbation trials. Therefore, the median of three predicted and unpredicted forward perturbation trials was calculated for each participant. Three trials were excluded due to a stepping response.

### 2.4 Statistical analysis

Statistical analysis was performed using SPSS statistical package (Version 24, SPSS statistics, IBM Corp. Armonk, NY, U.S.A) and Microsoft Excel (Microsoft Corporation, 2018). Normality of the data was determined using QQ plots, histogram and Shapiro Wilks test. In order to perform a Mann-Whitney *U* test, the data from both groups were automatically ordered in ascending fashion and ranked between 1 and 68. The distributions of the ranks were assessed by visual inspection of a population pyramid (histogram) produced in SPSS (Supplementary Figs. 1 & 2). Since the distributions of the two groups of the independent variable were not the same (i.e. different shapes), the Mann-Whitney *U* test was used to determine if there were statistically significant differences in the mean ranks of the dependent variable (mean integrated angular displacements, SF-36, ODI and HADS) between LDD pain and LDD no pain groups [38]. Higher and lower mean ranks described higher or lower total joint displacements, respectively. Effect sizes were also computed ($r=\frac{z}{\sqrt{\text{N}}\;}$, where z is z score and N is total number of observations). Spearman’s rho correlations were used to explore associations between mean integrated angular displacements, BMI and pain (NRS). Results were considered significant at P < 0.05 for all tests. Missing data were excluded case wise from the analysis and was not replaced by imputed values.

## 3 Results

Two groups were identified LDD pain (n = 34) and LDD no pain (n = 34). Age was not statistically significant between groups (p = 0.35). BMI was significantly higher in the LDD pain group than the LDD no pain group (Table 2). However, BMI did not correlate with significant kinematic findings (p = 0.06−0.96). In addition, there was no change in NRS scores for 100 % of participants before, during or after the perturbation trial.

**Table 2:** Participant demographics.

Groups	Age (years)	BMI (kg/m^−2^)	Gender
LDD pain	51.97 (11.90)	29.04 (6.12)	19 male, 15 female
LDD no pain	49.27 (11.73)	24.53 (2.91)	16 male, 18 female

### 3.1 Lower limb strategy

**Fig. 4:**
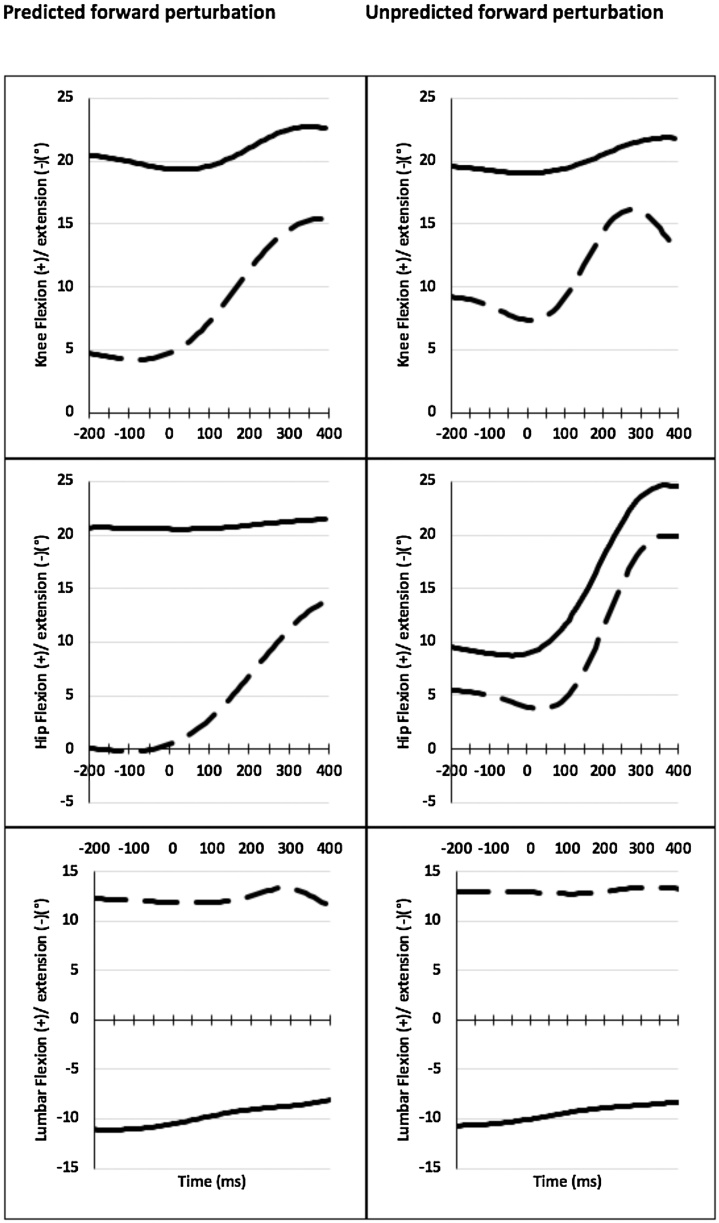
Representative traces of knee, hip and lumbar displacements from one patient (solid line) and healthy control (dashed line) during predicted and unpredicted forward perturbations. This figure represents the original kinematic data from the left knee (top panel), left hip (middle panel) and lumbar spine (bottom panel) of one LDD pain patient (solid line) and one LDD no pain control (dashed line) (°) following perturbation at time =0 ms. Patients use different hip, knee and lumbar strategies to healthy controls in the predicted scenario. In the unpredicted condition, although knee strategies remain different between groups, the hip and lumbar strategies appear similar. This indicates that the hip and lumbar strategies are most affected by anticipation of the perturbation event.

In the predicted condition, integrated hip and knee displacements were smaller in the LDD pain group (mean ranks: left hip APA2 = 27.85, CPA1 = 28.61, right hip APA2 = 28.85, left knee CPA2 = 30) than the LDD no pain group (mean ranks: left hip APA2 = 39.15, CPA1 = 38.39, right hip APA2 = 38.15, left knee CPA2 = 37) (U = 325–391, z=-2.51 - -1.97, r= −0.31 to −0.24, p = 0.049−0.04). In the unpredicted condition, smaller integrated knee displacements were similarly observed in the LDD pain group (mean rank: left knee CPA2 = 27.80) when compared with the LDD no pain group (mean rank: left knee CPA2 = 37.50) (U = 356, z=-2.08, r=-0.26, p = 0.04) (Fig. 4 & Supplementary Fig. 1).

Overall, this corresponded to a significant difference in anticipatory and compensatory lower limb strategy between groups in response to predicted and unpredicted forward perturbations.

### 3.2 Spinal strategy

In the predicted condition, there was no significant difference between groups in the APA phases (p>0.05). However, in the CPA phases, lumbar displacement was notably higher in the LDD pain group (mean ranks: lumbar CPA1 = 36.20, CPA2 = 34.60) than the LDD no pain group (mean ranks: lumbar CPA1 = 23.59, CPA2 = 25.24) (U = 249–297, z=-2.09−2.82, r = 0.27−0.37, p = 0.040−0.005). In the unpredicted condition, there was no significant differences in spinal segment displacement during the APA and CPA phases (p = 0.28−0.89).

These findings corresponded to a significant difference in compensatory lumbar strategy between groups in response to predicted forward perturbation with no significant difference in the unpredicted condition (Fig. 4 & Supplementary Fig. 2).

### 3.3 Self-reported clinical outcomes

In the predicted condition, there was a significant negative correlation observed between bilateral hip APAs and self-reported pain (NRS) (left hip APA2, r_s_=-0.43, p = 0.01, right hip APA2, r_s_=-0.35, p = 0.04). There were no significant correlations in the unpredicted condition (p > 0.05).

Mean ranks for depression, anxiety and ODI were significantly higher for the LDD pain group (mean ranks = 39.25–51.31) than the LDD no pain group (mean ranks 17.69–24.80) (U = 713–1150, z = 3.29–7.30, r = 0.41−0.88, p = 0.001). The LDD pain group had significantly lower mean ranks in terms of quality of life (mean ranks = 16.73–23.60), when compared to the LDD no pain group (mean ranks = 39.64–45.88) (U = 16.73–23.60, z=-6.46 to -3.50, r=-0.81 to -0.44, p = 0.0001). This indicated that the LDD pain group experienced higher levels of depression, anxiety and disability and lower quality of life than the LDD no pain group.

## 4 Discussion

The evaluation of kinematic strategy is frequently described in the literature when investigating differences between LBP and healthy controls during functional tasks [[7], [8], [9], [10], [11], [12]]. However, to our knowledge, this is the first time that a comprehensive evaluation of spinal and bilateral lower limb kinematic strategy has been described in relation to a specific LBP cohort. Using a novel marker set [26,27] and bespoke postural perturbations simulating public transport [34] it was possible to determine differences in kinematic strategy between LDD pain and LDD no pain groups. These differences extended beyond the biomechanical realm; significant differences in anxiety, depression, disability and quality of life were also observed between groups.

Healthcare professionals routinely use gait and STS tasks to assess LDD patients [39] as these tasks are important to patients and affect quality of life [8,10,39,40]. For this reason, prior to this current study, a preliminary assessment of peak angular displacements was undertaken in the frontal, sagittal and transverse plane during gait and STS tasks. There were significant differences in hip abduction and pelvic obliquity during gait (hip abduction and pelvic obliquity were lower in the LDD pain group when compared to the LDD no pain group, P < 0.05). However, there were no significant differences in joint displacement in transverse or sagittal planes during gait and no significant differences in any plane during the STS task. In agreement with previous research [9,12], these preliminary findings confirmed that in order to detect differences in kinematic strategy within an LDD cohort it would be necessary to consider more destabilising tasks. Since preliminary assessment of trunk and lower limb muscle activation established that significant differences could be reliably determined between LDD pain and LDD no pain groups using predictable and unpredictable forward perturbations within the sagittal plane [41], it seemed appropriate to use the same perturbation task to determine kinematic differences between these groups.

A recent systematic review, comparing anticipatory and compensatory responses to postural perturbation, found a lack of conclusive evidence to support kinematic differences between people with or without chronic LBP [8]. Definitive conclusions could not be made due to the reported heterogeneity of LBP samples and scarcity of high quality studies in this area, highlighting the need to explore such differences within a larger and well-defined cohort such as LDD.

It is proposed that stereotyped lower limb displacements enhance postural recovery during mechanical perturbations on a flat surface [42]. However factors, such as perturbation predictability and environmental factors, require a spectrum of mixed postural strategies [43]. In health, the ‘hip’ and ‘knee’ strategy are commonly observed, efficient, multi-segmental responses to perturbation at higher accelerations [44,45]. However, to circumvent a multi-segmental response, LBP patients tend to exhibit a reduced ‘hip strategy’, utilising reduced hip displacements for balance control when compared with healthy controls [43]. In the same way, the LDD pain group in this current study utilised reduced hip and knee displacements when compared with LDD no pain group during the APA and CPA phases of predicted and unpredicted forward perturbation. This maladaptive sagittal postural response or protective strategy [43,46] may be secondary to altered muscle control, proprioception [43] and reflect the high levels of anxiety demonstrated by the LDD pain group in this study.

Although motor control impairments of the lower limb are typically described, significant differences in spinal strategy are documented. Healthy adults appear more adaptable than LBP patients, using an unrestricted repertoire of multiple joint segment motion in response to perturbation [15,[46], [47], [48]]. However, LBP patients appear to exhibit consistently larger lumbar and smaller lower limb displacements than healthy controls in response to postural perturbation [48,49]. This concurs with this study, in which smaller lower limb displacements accompanied larger compensatory lumbar movements in the LDD pain group during predicted perturbation while the LDD no pain group experienced the opposite effect. This finding is of interest since the LDD pain group exhibited increased compensatory displacement in the same region in which LDD was confirmed on MRI.

Although, we cannot presume a causal effect, it seems that such differences in the postural chain are more likely to be uncovered through the comprehensive examination of both the spine and lower limbs. Since the postural strategy adopted by the LDD pain group involved joints proximal (lumbar) and distal (hip and knee) to the site of reported LBP, this may reflect a centrally mediated change or a change within the nervous system associated with task prediction [50]. This requires further investigation.

In response to unpredictable perturbations there was no difference in lumbar and hip displacements between groups. These findings are noteworthy for two reasons. Firstly, although ‘trunk stiffening’ has been reported to result from similarly unpredictable perturbation scenarios in LBP patients [50], a recent systematic review agrees that there is a lack of convincing evidence to support this [51]. Secondly, it has been shown that LBP patients move differently due to the anticipation or avoidance of pain provoking postures [52]. Therefore, it seems unsurprising that when the condition is unpredictable there is no strategic difference between groups; they both move in the same way.

A strength of this study was that the a priori sample size estimate was exceeded (34 per group). In addition, potential confounders such as age, sex, BMI and task related pain changes were not found to influence results. However, the ‘Imperial Spine’ marker set [26,27] was used to evaluate sagittal kinematics with several accepted assumptions. Firstly, the trunk and bilateral lower limbs were assumed to consist of rigid segments. Secondly, it was assumed that skin mounted markers reflected the motion of underlying bone, despite inherent motion artefact [53]. It is also acknowledged that experimentation using different perturbation types (accelerations, directions or surfaces), outcomes (temporal features and kinetics) and functionals tasks could be used to expose additional deficits. Since causality cannot be implied from this observational study, further longitudinal studies will be required.

### 4.1 Clinical relevance

In this study it was observed that significant differences in kinematic strategy are not exclusive to the trunk but also occur in the lower limbs. This suggests that a comprehensive examination of the trunks and bilateral lower limbs of LDD patients is required in clinical research and practice, in order to prevent the potential underestimation of motor control deficits.

The kinematic differences observed between the LDD pain and LDD no pain groups in this study appear similar to those previously reported between people with LBP (for whom the cause of LBP has not been determined) and healthy controls [48,49]. Understanding that the specific motor control phenotypes that LDD patients express are similar to those expressed by people with undiagnosed LBP suggests that a similarly targeted motor retraining approach, which demonstrates the potential to change motor control strategies [[54], [55], [56], [57]], could prove beneficial for both. However, as this current study demonstrates, management will need to extend beyond the biomechanical realm in order to address high levels of anxiety, depression, disability and diminished quality of life experienced by LDD patients.

Correlation analysis in symptomatic LDD patients revealed a negative correlation between bilateral hip APAs and self-reported pain (NRS) in the predicted condition. It is of note that, in the same predicted condition, the same parameters (left and right hip APA2) were also observed to be significantly different between the LDD pain and LDD no pain groups. A recent systematic review highlighted that the effect of LBP on APAs and CPAs is unclear, with authors proposing a link between impaired limb motor control and the risk of injury proximal or distal to the affected trunk or lower limb segments [8]. Therefore, assuming that a NRS sufficiently reflects the pain experienced by patients, this current study provides further insight. The observed negative correlation suggests that increasing levels of pain are associated with decreasing anticipatory hip displacement or reduced ‘hip strategy’, which may reflect a change in the ‘central set’ or the preparatory state of the CNS in response to predictable perturbations [50]. Since APAs occur prior to a predicted perturbation event in order to minimise disequilibrium or falling [8,36], a reduced anticipatory response, may represent a higher risk of falling in LDD patients.

## 5 Conclusions

Symptomatic LDD patients exhibit different kinematic strategies to their asymptomatic LDD counterparts. Strategic differences are dependent upon task predictability and are not restricted to the lumbar region, confirming the need for future 3DMC studies to use marker sets, such as the ‘Imperial Spine’, which include bilateral lower limb segments. Differences in functional ability, mental health and quality of life also reflect underlying complexity. Therefore, effective rehabilitation for symptomatic LDD patients will require a multi-faceted approach. Evaluation of spine and lower limb kinematics under destabilising conditions, in addition to self-reported clinical outcomes, may prove a better indicator of LBP and/or risk of recurrence than LDD itself.

## Declaration of Competing Interest

The authors report no declarations of interest.

## References

[1] Todd N.V. (2017). The surgical treatment of non-specific low back pain. Bone Joint J..

[2] Dechartres A., Trinquart L., Boutron I., Ravaud P. (2013). Influence of trial sample size on treatment effect estimates: meta-epidemiological study. BMJ.

[3] Koes B., van Tulder M., Lin C.-W., Macedo L., McAuley J., Maher C. (2010). An updated overview of clinical guidelines for the management of non-specific low back pain in primary care. Eur. Spine J..

[4] Christe G., Redhead L., Legrand T., Jolles B.M., Favre J. (2016). Multi-segment analysis of spinal kinematics during sit-to-stand in patients with chronic low back pain. J. Biomech..

[5] Pourahmadi M.R., Ebrahimi Takamjani I., Jaberzadeh S., Sarrafzadeh J., Sanjari M.A., Bagheri R. (2018). Test-retest reliability of sit-to-stand and stand-to-sit analysis in people with and without chronic non-specific low back pain. Musculoskelet. Sci. Pract..

[6] Svendsen J.H., Svarrer H., Laessoe U., Vollenbroek-Hutten M., Madeleine P. (2013). Standardized activities of daily living in presence of sub-acute low-back pain: a pilot study. J. Electromyogr. Kinesiol..

[7] Seay J.F., Van Emmerik R.E., Hamill J. (2011). Influence of low back pain status on pelvis-trunk coordination during walking and running. Spine.

[8] Knox M.F., Chipchase L.S., Schabrun S.M., Romero R.J., Marshall P.W.M. (2018). Anticipatory and compensatory postural adjustments in people with low back pain: a systematic review and meta-analysis. Spine J..

[9] Gombatto S.P., Brock T., DeLork A., Jones G., Madden E., Rinere C. (2015). Lumbar spine kinematics during walking in people with and people without low back pain. Gait Posture.

[10] Livshits G., Popham M., Malkin I., Sambrook P.N., MacGregor A.J., Spector T. (2011). Lumbar disc degeneration and genetic factors are the main risk factors for low back pain in women: the UK Twin Spine Study. Ann. Rheum. Dis..

[11] Brinjikji W., Diehn F.E., Jarvik J.G., Carr C.M., Kallmes D.F., Murad M.H. (2015). MRI findings of disc degeneration are more prevalent in adults with low back pain than in asymptomatic controls: a systematic review and meta-analysis. AJNR Am. J. Neuroradiol..

[12] Hartvigsen J., Hancock M.J., Kongsted A., Louw Q., Ferreira M.L., Genevay S. (2018). What low back pain is and why we need to pay attention. Lancet (London, England)..

[13] Cholewicki J., Silfies S.P., Shah R.A., Greene H.S., Reeves N.P., Alvi K. (2005). Delayed trunk muscle reflex responses increase the risk of low back injuries. Spine..

[14] Jacobs J.V., Horak F.B. (2007). Cortical control of postural responses. J. Neural Transm..

[15] Mok N.W., Hodges P.W. (2013). Movement of the lumbar spine is critical for maintenance of postural recovery following support surface perturbation. Exp. Brain Res..

[16] Belen’kii V.E., Gurfinkel V.S., Pal’tsev E.I. (1967). [Control elements of voluntary movements]. Biofizika.

[17] Bouisset S., Zattara M. (1987). Biomechanical study of the programming of anticipatory postural adjustments associated with voluntary movement. J. Biomech..

[18] McIlroy W.E., Maki B.E. (1996). Age-related changes in compensatory stepping in response to unpredictable perturbations. The journals of gerontology Series A. Biol. Sci. Med. Sci..

[19] Nashner L.M., McCollum G. (1985). The organization of human postural movements: a formal basis and experimental synthesis. Behav. Brain Sci..

[20] Cheung K.M.C., Karppinen J., Chan D., Ho D., WH, Song Y.-Q., Sham P., Cheah K.S.E., Leong J.C.Y., Luk K.D.K. (2009). Pattern of lumbar magnetic resonance imaging changes in a population study of one thousand forty-three individuals. Spine.

[21] Takatalo J., Karppinen J., Taimela S., Niinimäki J., Laitinen J., Sequeiros R.B. (2013). Association of abdominal obesity with lumbar disc degeneration – a magnetic resonance imaging study. PLoS One.

[22] Dworkin R.H., Turk D.C., Farrar J.T., Haythornthwaite J.A., Jensen M.P., Katz N.P. (2005). Core outcome measures for chronic pain clinical trials: IMMPACT recommendations. Pain.

[23] Jenkinson C., Stewart-Brown S., Petersen S., Paice C. (1999). Assessment of the SF-36 version 2 in the United Kingdom. J. Epidemiol. Community Health.

[24] Roland M., Fairbank J. (2000). The Roland-Morris disability questionnaire and the Oswestry disability questionnaire. Spine.

[25] Zigmond A.S., Snaith R.P. (1983). The hospital anxiety and depression scale. Acta Psychiatr. Scand..

[26] Deane J.A., Papi E., Phillips A.T.M., McGregor A.H. (2018). The design and development of the ‘Imperial Spinal Model’: a holistic and reliable approach to assessing kinematics using 3D motion technology. Bone Joint J..

[27] Deane J.A., Papi E., Phillips A.T.M. (2020). Reliability and minimal detectable change of the ‘Imperial Spine’ marker set for the evaluation of spinal and lower limb kinematics in adults. BMC Res. Notes.

[28] Borhani M., McGregor A.H., Bull A.M. (2013). An alternative technical marker set for the pelvis is more repeatable than the standard pelvic marker set. Gait Posture.

[29] Ugbolue U.C., Papi E., Kaliarntas K.T. (2013). The evaluation of an inexpensive, 2D, video based gait assessment system for clinical use. Gait Posture.

[30] Wu G., Siegler S., Allard P., Kirtley C., Leardini A., Rosenbaum D. (2002). ISB recommendation on definitions of joint coordinate system of various joints for the reporting of human joint motion—part I: ankle, hip, and spine. J. Biomech..

[31] Papi E., Ugbolue U.C., Solomonidis S.E., Rowe P.J. (2011). Proceedings of Twenty Third International Society of Biomechanics Congress.

[32] Harrington M.E., Zavatsky A.B., Lawson S.E.M., Yuan Z., Theologis T.N. (2007). Prediction of the hip joint centre in adults, children, and patients with cerebral palsy based on magnetic resonance imaging. J. Biomech..

[33] Merriaux P., Dupuis Y., Boutteau R., Vasseur P., Savatier X. (2017). A study of vicon system positioning performance. Sensors (Basel, Switzerland).

[34] Favier C., Deane J., McGregor A., Phillips A.T.M. (2019). Design and preliminary testing of a low-cost balance perturbation system for the evaluation of real life postural adjustment on public transport. J. Med. Eng. Technol..

[35] Allison G.T. (2003). Trunk muscle onset detection technique for EMG signals with ECG artefact. J. Electromyogr. Kinesiol..

[36] Santos M.J., Kanekar N., Aruin A.S. (2010). The role of anticipatory postural adjustments in compensatory control of posture: 2. Biomechanical analysis. J. Electromyogr. Kinesiol..

[37] Cavanagh P.R., Komi P.V. (1979). Electromechanical delay in human skeletal muscle under concentric and eccentric contractions. Eur. J. Appl. Physiol. Occup. Physiol..

[38] Hart A. (2001). Mann-Whitney test is not just a test of medians: difference in spread can be important. BMJ.

[39] Deane J.A., McGregor A.H. (2016). Current and future perspectives on lumbar degenerative disc disease: a UK survey exploring specialist multidisciplinary clinical opinion. BMJ Open.

[40] Pourahmadi M.R., Takamjani I.E., Jaberzadeh S., Sarrafzadeh J., Sanjari M.A., Bagheri R. (2017). Kinematics of the spine during sit-to-Stand using motion analysis systems: a systematic review of literature. J. Sport Rehabil..

[41] Deane J.A., Lim A.K.P., Phillips A.T.M., Strutton P.H., McGregor A.H. (2019). Why do only some people with lumbar disc degeneration have recurrent low back pain? An examination of postural control strategy. Bone Joint J..

[42] Horak F.B., Nashner L.M. (1986). Central programming of postural movements: adaptation to altered support-surface configurations. J. Neurophysiol..

[43] Mok N.W., Brauer S.G., Hodges P.W. (2004). Hip strategy for balance control in quiet standing is reduced in people with low back pain. Spine.

[44] Runge C.F., Shupert C.L., Horak F.B., Zajac F.E. (1999). Ankle and hip postural strategies defined by joint torques. Gait Posture.

[45] Kuo A.D. (1995). An optimal control model for analyzing human postural balance. IEEE Trans. Biomed. Eng..

[46] Lin Y.C., Niu C.C., Nikkhoo M., Lu M.L., Chen W.C., Fu C.J., Cheng C.H. (2018). Postural stability and trunk muscle resposnes to the static and perturbed balance tasks in individuals with and without symptomatic degenerative lumbar disease. Gait Posture.

[47] Sperry M.M., Phillips A.T.M., McGregor A.H. (2019). Lower back pain and healthy subjects exhibit distinct lower limb perturbation response strategies: a preliminary study’. J. Back Musculoskelet. Rehabil..

[48] Mok N.W., Brauer S.G., Hodges P.W. (2007). Failure to use movement in postural strategies leads to increased spinal displacement in low back pain. Spine.

[49] Sung P.S., Danial P. (2018). Trunk reaction time and kinematic changes following slip perturbations in subjects with recurrent low back pain. Ann. Biomed. Eng..

[50] Jones S.L., Henry S.M., Raasch C.C., Hitt J.R., Bunn J.Y. (2012). Individuals with non-specific low back pain use a trunk stiffening strategy to maintain upright posture. J. Electromyogr. Kinesiol..

[51] Prins M.R., Griffioen M., Veeger T.T.J., Kiers H., Meijer O.G., van der Wurff P. (2018). Evidence of splinting in low back pain? A systematic review of perturbation studies. Eur. Spine J..

[52] Moseley G.L., Nicholas M.K., Hodges P.W. (2004). Does anticipation of back pain predispose to back trouble?. Brain.

[53] Leardini A., Chiari L., Croce U.D., Cappozzo A. (2005). Human movement analysis using stereophotogrammetry: part 3. Soft tissue artifact assessment and compensation. Gait Posture.

[54] Tsao H., Druitt T.R., Schollum T.M., Hodges P.W. (2010). Motor training of the lumbar paraspinal muscles induces immediate changes in motor coordination in patients with recurrent low back pain. J. Pain.

[55] Knox M.F., Chipchase L.S., Schabrun S.M., Marshall P.W.M. (2017). Improved compensatory postural adjustments of the deep abdominals following exercise in people with chronic low back pain. J. Electromyogr. Kinesiol..

[56] Arampatzis A., Schroll A., Moreno Catalá M. (2017). A random-perturbation therapy in chronic non-specific low-back pain patients: a randomised controlled trial. Eur. J. Appl. Physiol..

[57] Muthukrishnan R., Shenoy S.D., Jaspal S.S., Nellikunja S., Fernandes S. (2010). The differential effects of core stabilization exercise regime and conventional physiotherapy regime on postural control parameters during perturbation in patients with movement and control impairment chronic low back pain. Sport. Med. Arthrosc. Rehabil. Ther. Technol..

