# Supplementary material for: Symptomatic individuals with Lumbar Disc Degeneration use different anticipatory and compensatory kinematic strategies to asymptomatic controls in response to postural perturbation
Source: Gait Posture. 2022 May;94:222–9. doi: 10.1016/j.gaitpost.2021.03.037 (PMC9099249; doi:10.1016/j.gaitpost.2021.03.037)
Supplement: Supplementary file 1 [file mmc1.docx]

Supplementary Fig. 1. Distribution of lower limb ranks for LDD pain and LDD no pain groups in the CPA2 phase of predicted and unpredicted forward perturbation

The distribution of ranks of integrated left knee (LKnee) displacement during the CPA2 phase, for LDD pain groups (right, grey) and LDD no pain group (left, white). In the CPA2 phase of predicted (top panel) and unpredicted perturbation (bottom panel), the LDD pain group (26.85, 27.80) exhibited lower mean ranks than the LDD no pain group (38.52, 37.50). This suggests that the LDD pain group exhibit smaller integrated knee displacements in the sagittal plane than the LDD no pain group during the CPA2 phase of predicted and unpredicted perturbation. N=total number of participants in each group. The frequency and LKnee CPA2 (integral in arbitrary units) are presented on the X and Y axis respectively. The CPA2 phase represents 150ms (250-400ms following perturbation).

Supplementary Fig. 2. Distribution of ranks for ‘LDD no pain’ and ‘LDD pain’ groups in the CPA1 phase of predicted forward perturbation.

The distribution of ranks of integrated lumbar (Lum) displacement during the CPA1 phase for LDD no pain group (left, white) and LDD pain groups (right, grey). In the CPA1 phase of predicted forward perturbation, the LDD pain group exhibit a higher mean rank (36.20) than the LDD no pain group (23.59). This suggests that the LDD pain group exhibit larger integrated lumbar displacements in the sagittal plane than the LDD no pain group during the CPA1 phase of predicted perturbation. In the unpredicted condition there is no significant difference in mean ranks between LDD pain (30.53) and LDD no pain (29.48) groups. N=total number of participants in each group. The frequency and LumCPA1 (integral in arbitrary units) are presented on the X and Y axis respectively. The CPA1 phase represents 150ms (100-250ms following perturbation).
