# Supplementary figures and images for: Symptomatic individuals with Lumbar Disc Degeneration use different anticipatory and compensatory kinematic strategies to asymptomatic controls in response to postural perturbation
Source: Gait Posture. 2022 May;94:222–9. doi: 10.1016/j.gaitpost.2021.03.037 (PMC9099249; doi:10.1016/j.gaitpost.2021.03.037)

## Slide 1
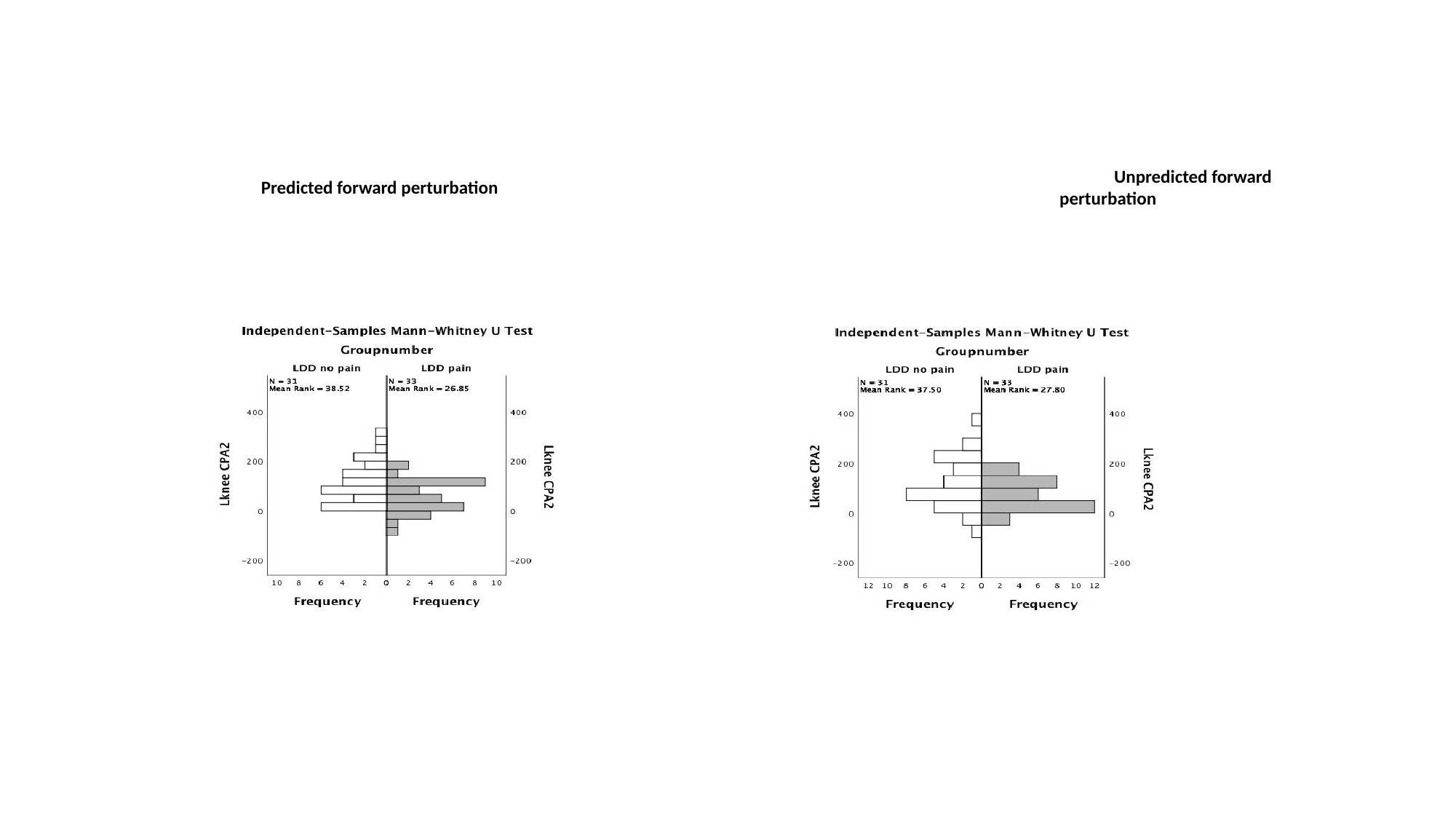

Predicted forward perturbation
Unpredicted forward perturbation

Supplement: Supplementary file 2 [file mmc2.pptx]

## Slide 1
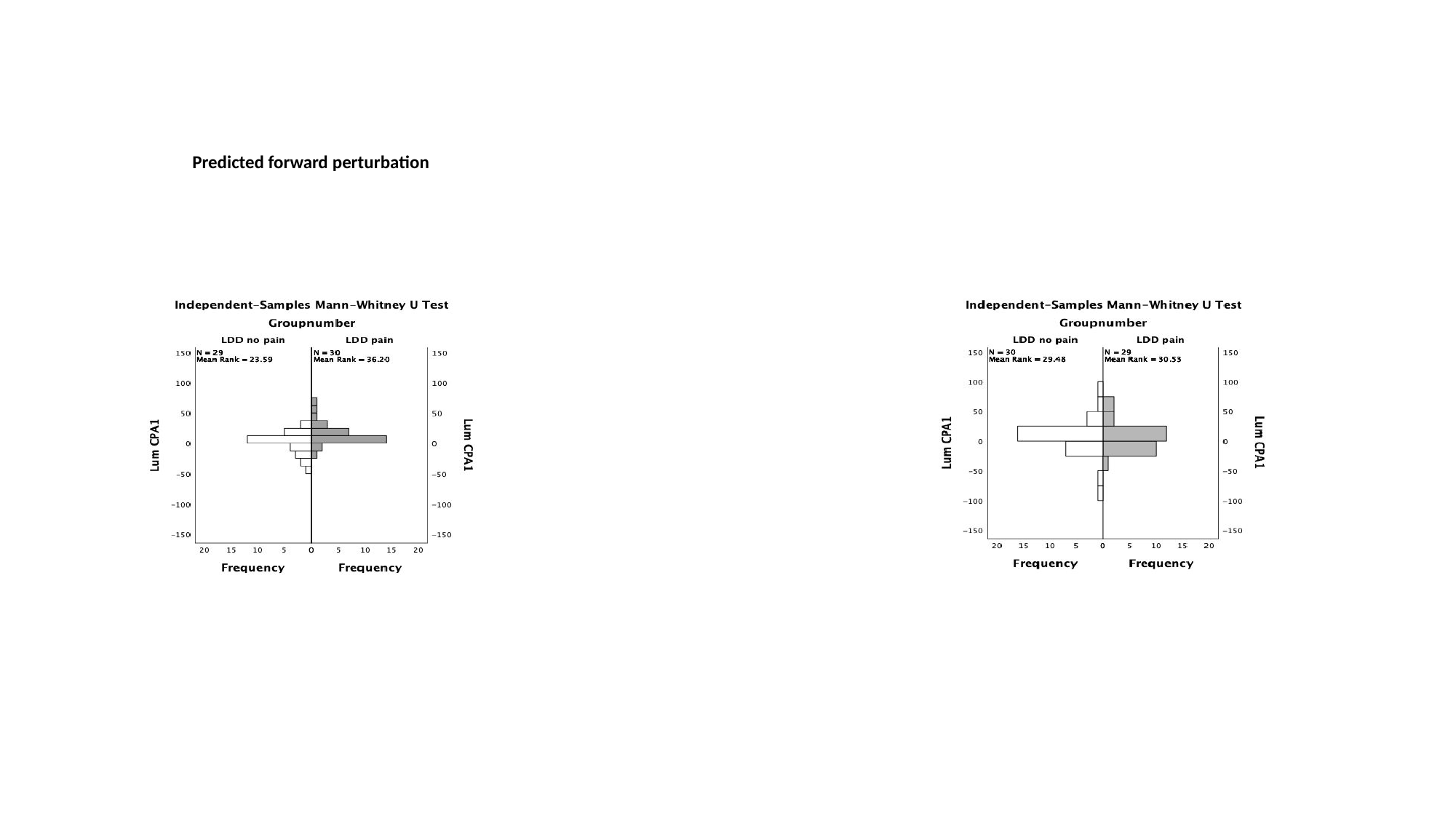

Predicted forward perturbation
Unpredicted forward perturbation

Supplement: Supplementary file 3 [file mmc3.pptx]
